# Detection of Antiseptic-Resistance Genes in *Pseudomonas* and *Acinetobacter* spp. Isolated From Burn Patients

**DOI:** 10.17795/jjnpp-15402

**Published:** 2014-04-03

**Authors:** Mohammadreza Mahzounieh, Sheida Khoshnood, Azizollah Ebrahimi, Saeid Habibian, Maryam Yaghoubian

**Affiliations:** 1 Department of Pathobiology, Faculty of Veterinary Medicine, Shahrekord University, Shahrekord, IR Iran

**Keywords:** *Pseudomonas aeruginosa*, *Acinetobacter*, Quaternary Ammonium Compounds

## Abstract

**Background:**

Quaternary ammonium compounds (QAC), which contain benzalkonium chloride as the most widely used agent, are employed as wound and skin antiseptics, as well as disinfectants in hospitals. The resistance mechanism to disinfectants is usually determine by genes which are related to resistance to quaternary ammonium compounds, namely, *qac*E, *qac*ΔE1, *qac*ΔE1 that are found in Gram-negative bacteria.

**Objectives:**

The aim of this study was to determine the incidence of antiseptic resistance genes, *qac*E and *qac*ΔE1, in clinical isolates of *Pseudomonas aeruginosa* and *Acinetobacter bumanii*.

**Materials and Methods:**

In this study, 83 clinical isolates of *Pseudomonas aeruginosa*, and 5 isolates of *Acinetobacter baumannii* from burn hospitals in Tehran and Isfahan provinces in 2010-2011, were tested by the PCR method.

**Results:**

Out of the 83 clinical isolates of *Pseudomonas aeruginosa*, 49 isolates (50%) had the *qac*E gene, and 76 isolates (91.5%) had the *qac*ΔE1 gene. In addition, in 5 isolates of* Acinetobacter bumanii*, 2 isolates (40%) had the *qac*E gene, and 4 isolates (80%) had the *qac*ΔE1 gene.

**Conclusions:**

This study shows that the genes which harbored resistance to quaternary ammonium compound antiseptics are widespread among *Pseudomonas aeruginosa* and *Acinetobacter bumanii *isolates in burn patients.

## 1. Background

Hospital acquired infections are important because they cause disease, economical loss and death in hospital inpatients. These infections are difficult to treat and sometimes cause death. Thus, they count as a serious and growing risk factor that threatens the health of almost all patients who are confined in hospitals (1). Patients, who have burn injuries, are at increased risk of hospital acquired infections, because burn wounds are suitable places to grow opportunistic bacteria, including *Pseudomonas* and *Acinetobacter* (2). *Pseudomonas aeruginosa* is a Gram-negative, obligate aerobic bacterium, and the most prevalent factor in hospital related infections include: pneumonia, bacteremia and serious infections in burn patients. This bacterium is ubiquitous and originates in areas such as water, soil and skin (3-5).

*Acinetobacter baumannii* is one of the most widespread bacteria found among hospital acquired infections, and it usually has a second/third rank among the prevalent pathogens of these kinds of infections (5-7). Infection with this bacterium is very dangerous, particularly for patients who are confined in hospitals’ intensive care units (5, 8).* Acinetobacter* may cause infection in the respiratory system, urinary tract and meningitis (5, 9). Various types of antiseptics are utilized in hospitals and medical centers, and until now many samples of bacteria resistant to antiseptics have been reported. Resistance related to *qac,* and small multidrug resistance (SMR) genes are due to resistance against DNA-intercalating dyes (like ethidium bromide) and quaternary ammonium compounds (like benzalkonium chloride), therefore, resistance mechanisms are coded by *smr*, *qac*E and *qacA*. These gene products are transmembrane proteins (10). Although the most frequent genes which code *smr*, *qac*E, *qacA*, and *qacF* are found in Gram-positive bacteria, only three types of these genes have been determined in Gram-negative bacteria. The *qac*E gene (including its attenuated variant *qac*EΔ1) is widespread in Gram-negative bacteria, mainly in Enterobacteriaceae and *Pseudomonas *spp. because these genes are located in class 1 integrons, which in Gram-negative bacteria commonly harbor *qac*EΔ1 (11). The *qac*ΔE1 gene is a mutation of the *qac*E gene, which acts as a multidrug transfer gene (12). Although the *qac* genes were named after one of their main substrates (QACs) was found, the spectrum of their activity is much broader. More than 30 lipophilic cationic compounds belonging to at least 12 different chemical classes are recognized as targets of *qac*-mediated resistance (13).

In this study, we determined the distribution of the *qac*E and *qac*ΔE1 genes in *Pseudomonas aeruginosa* and *Acinetobacter baumannii* isolated from clinical specimens using PCR.

## 2. Objectives

The aim of this study was to determine the distribution of antiseptic resistance genes *qac*E and *qac*ΔE1 among the clinical isolates of *Pseudomonas aeruginosa* and *Acinetobacter* *bumanii* found in burn patients.

## 3. Materials and Methods

### 3.1. Source of Isolates

A total of 83 clinical isolates of *Pseudomonas aeruginosa*, and five isolates of *Acinetobacter baumannii* were collected from burn hospitals in Tehran and Isfahan provinces, during 2010-2012. All bacteria were identified according to colony characteristics, bacterial morphology, Gram-negative staining and the results of routine biochemical tests. Most of the *P. aeroginosa* isolates produced the blue pigment pyocyanin.

### 3.2. Extraction of Total DNA

Isolates were grown in Luria-Bertani broth at 37˚C. The DNA was extracted from a fresh culture of each isolate in Luria-Bertani medium. One ml of bacterial suspension was boiled at 100˚C for 5 minutes in a water bath. Heat disrupted the bacterial cell walls, and the DNA was released into the medium. Finally, the tubes were centrifuged at 10 000 rpm for 5 minutes, and the supernatant was used for PCR (14). The quantity and quality of extracted nucleic acids were estimated by electrophoresis on 0.7% agarose gel.

### 3.3. Detection of Antiseptic-resistance Genes by PCR

For the detection of the specific sequence of the *qac*E gene by PCR, we used Taq DNA Polymerase Master Mix RED 2.0x (Ampliqon, Denmark), 12.5 µL, sterile distilled water, 8.5 µL, and 1 µL of each primer in a 25 µL reaction. Two µL of extracted DNA was added to each tube. Primers were synthesized by TAG (Copenhagen). The PCR was conducted with 35 cycles as follows: denaturation at 94˚C for 45 seconds*,* annealing at 55˚C for 45 seconds, and extension at 72˚C for 45 seconds in a thermocycler machine (ABI Geneamp 9700, USA). Preincubation at 94˚C for 3 minutes, and a final extension cycle at 72˚C for 8 minutes, were also included (11). The products of the PCR were detected by electrophoresis on a 1.5% agarose gel in a TAE buffer. The gels were stained with ethidium bromide and visualized by UV in a transilluminator (UVitec, UK) (Table 1). The PCR program for *qac*ΔE1 detection was carried out for one cycle at 93˚C for 2 minutes followed by 35 cycles which included: denaturation step at 94˚C for 30 seconds, annealing step at 55˚C for 30 seconds and extension step at 72˚C for one minute. A final extension step was done at 72˚C for 5 minutes at the end (11).

**Table 1.** Primer Sequences for Detection of Antiseptic Resistance Genes in *Pseudomonas aeruginosa* and *Acinetobacter bumanii*

Gene	Amplicone, bp
***qacE***	300
Forward: 5ˊATG AAA GGC TGG CTT3ˊ	
Reverse: 5ˊTCA CCA TGG CGT CGG3ˊ	
***QacΔE1***	335
Forward: 5ˊTAG CGA GGG CTT TAC TAA GC3ˊ	
Reverse: 5ˊATT CGA AAT GCC GAA CAC CG3ˊ	

## 4. Results

Genomic detection of *qac*E and *qac*ΔE1 showed that 49 (59%) and 76 (91.5%), out of 83 *Pseudomonas aeruginosa* isolates had the *qac*E and *qac*ΔE1 genes, respectively. Positive PCR products showed 300 bp and 335 bp amplicons, respectively. There were 2 (40%) and 4 (80%) isolates among the 5 *Acinetobacter baumannii* isolates, which had *qac*E and E1 genes, respectively. The results are summarized in Table 2.

**Table 2.** Incidence of Resistance Genes Related to Quaternary Ammonium Compounds Among Clinical Isolates of *Pseudomonas* and *Acinetobacter* ^a^

Gene	*Pseudomonas aeruginosa*		*Acinetobacter baumannii*	
	Positive Samples	Total	Positive Samples	Total
***qacE***	49 (59)	83	2 (40)	5
**E1**	76 (91.5)	83	4 (80)	5

^a^ Data are presented in No. (%).

## 5. Discussion

The skin is the first line of defense in the body which is exposed to the external environment. Burn injuries destroy the body's defense line integrity (skin), which naturally prevents localization and invasions of bacteria, fungi and viruses. According to previous records, more than 75% of the death toll that occurs after burn injuries is due to infections, which are caused by suppression of the immune system following damage to the skin caused by burns. These cases occur in inpatients suffering from skin trauma or immunosuppression. Long periods of stay in intensive care burn units, using vein cutters, urethral sounds and treatment with broad spectrum antibiotics, are also predisposing factors in these patients (15, 16). *Pseudomonas aeruginosa* is one of the opportunist human pathogens which contaminate various hospital wards. Many bacterial isolates produce exoenzymes and toxins which may damage the tissues of burn patients and cause numerous infections. Burn injuries are counted as one of the severe problems found in many parts of the world, particularly in developing countries. Pneumonia caused by *Pseudomonas aeruginosa*.

*Pseudomonas aeruginosa* is one of the most common Gram-negative bacteria in hospital acquired infections, and because it is naturally resistant to many drugs, it has the potential to be unresponsive to various effective antibiotics. As a result, contamination with these microorganisms is a regular and complicated problem for confined patients, especially burn patients (17). *Acinetobacter baumannii* is one of the non-fermenting Gram-negative bacteria which are commonly found in water and soil. This organism was sensitive to most antibiotics until 1970, but nowadays, it has become the second most widespread agent in hospital acquired infections around the world and many of the isolates are now resistant to commonly used antibacterial factors (18). The *qac*E and *qac*ΔE1 genes are commonly found in Gram-negative bacteria, because these genes are located in conserved sequences of integron class 1. Resistance genes related to *qacA*nd antibiotics are both carried by class 1 integrons, so it increases concerns about gene expression that is resistant to QAC, along with the increasing resistance to antibiotics by class 1 integrons.

Similarly, with the escalating problems of bacterial resistance to antibiotics in modern hospitals, bacterial resistance to antiseptics is also increasing. This subject has convinced physicians, that the problem of resistance to antibiotics could be concurrent with the abundant and irregular using of antiseptics over the long term, therefore, the treatment and control of nosocomial infections are difficult, expensive and sometimes even impossible (11). Quaternary ammonium compounds are used as antiseptics for the skin. Paulsen et al. reported that Gram-negative bacteria which harbored *qac*E and *qac*ΔE1 genes were resistant to intercalating colors and QAC (12). Corbella et al. reported that personnel, who had not had any contact with infected patients, carried *Acinetobacter* on their hands. This report implied the widespread presence of *Acinetobacter* in the hospital environment (8). In results of a study conducted by Meric et al. in Turkey, *Acinetobacter* was the second most widespread organism in the ICU, with a 26.5% incidence (18).

To control and prevent the spread of nosocomial infections with these two important bacteria, many commercial products based on ammonium quaternary compounds (QAC) are currently used in considerable quantities as antiseptic agents in hospitals and medical centers. Thus, recognition of effective antiseptics is a major concern for health care professionals. Infections with *Pseudomonas aeruginosa* and *Acinetobacter baumannii* are growing in increasing numbers and due to the intrinsic and chronic resistance of these bacteria to QAC, more preventive actions are necessary. Our results agreed with the results of the Kazama et al. study (1982-1995). They conducted their study on species of Gram-negative bacteria, and found the *qac*ΔE1 gene in 41 species and the *qac*E gene in 15 species of *Pseudomonas,* from a total of 63 clinical isolates of *Pseudomonas aeruginosa* (19). Kucken et al. determined that *qac*ΔE1 and *qac*E genes were present in 65.1% and 23.8% of 63 isolated *Pseudomonas,* respectively. According to our results, it seems that the incidence of *qac*ΔE1and *qac*E genes, which were reported by Kazama et al., are also starting to increase nowadays (11), therefore, further studies are necessary in order to find more effective antiseptics.

These results showed a high resistance to antiseptics and among these are the two important bacteria, *Pseudomonas* and *Acinetobacter,* which are commonly found in burn infections. It suggests that, antiseptics which contain benzalkonium chloride are not the correct agent to use in high risk hospital wards.
